# Supplementary figures and images for: Population genetics and phylogeographic history of the insular lizard Podarcis lilfordi (Gunther, 1874) from the Balearic Islands based on genome‐wide polymorphic data
Source: Ecol Evol. 2024 May 23;14(5):e11407. doi: 10.1002/ece3.11407 (PMC11116764; doi:10.1002/ece3.11407)

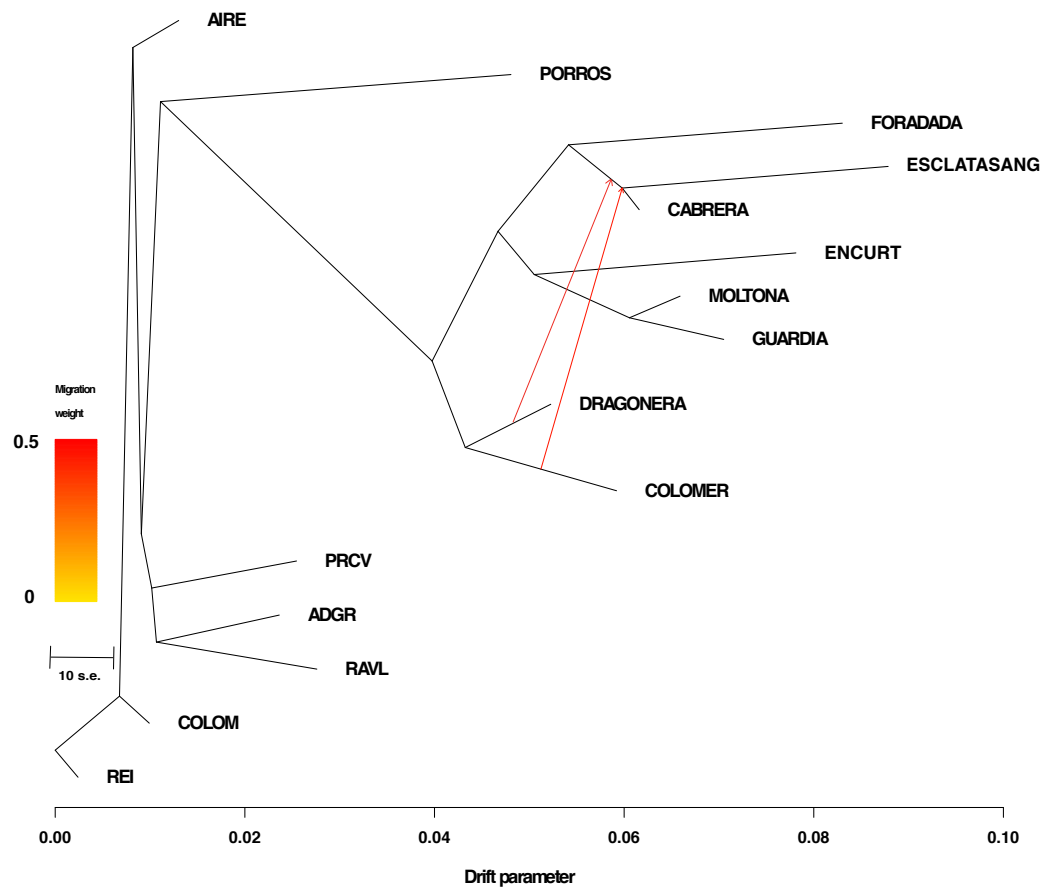

Supplement: Supplementary file 1 — Figure S1. [file ECE3-14-e11407-s004.pdf]

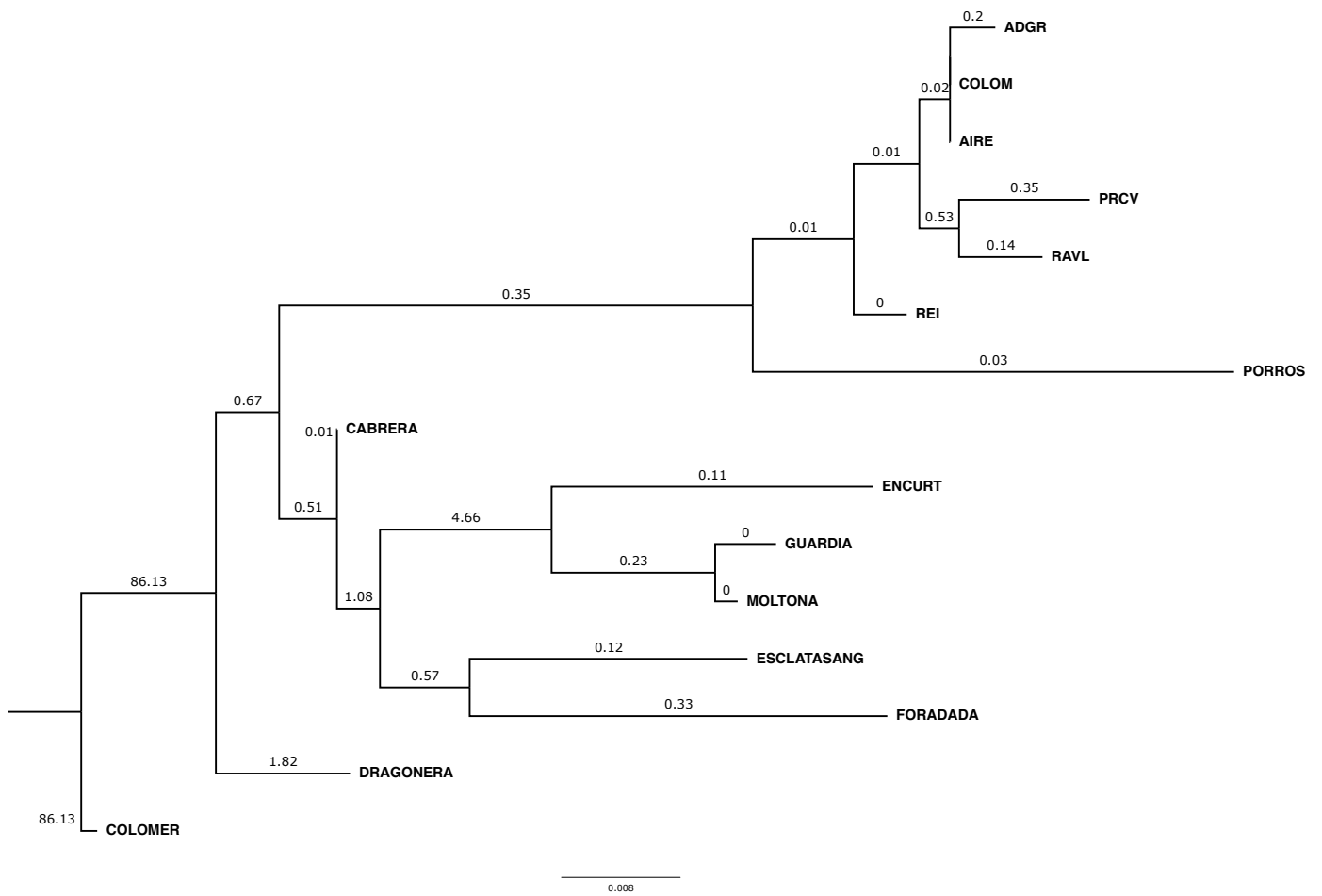

Supplement: Supplementary file 2 — Figure S2. [file ECE3-14-e11407-s002.pdf]

A)

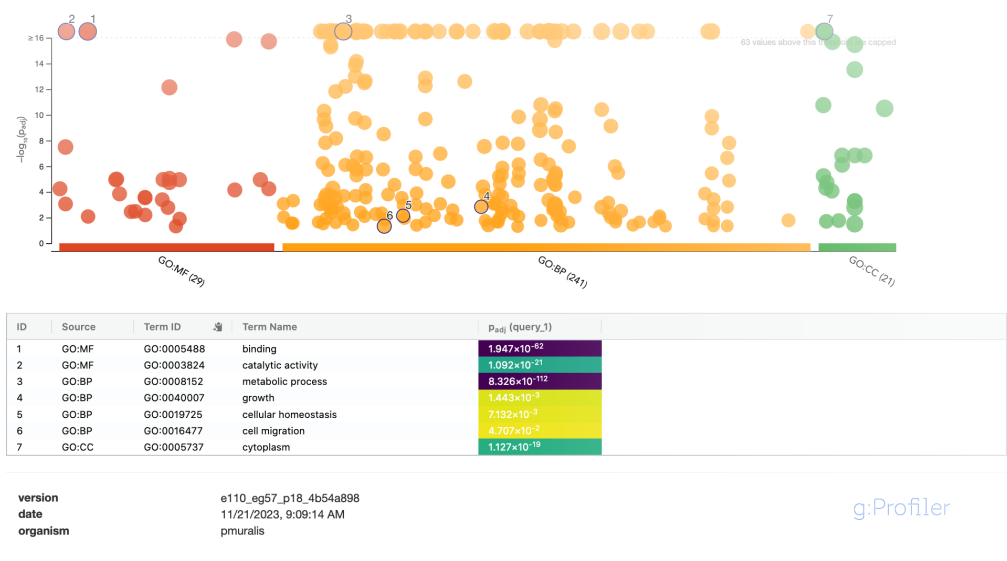

B)

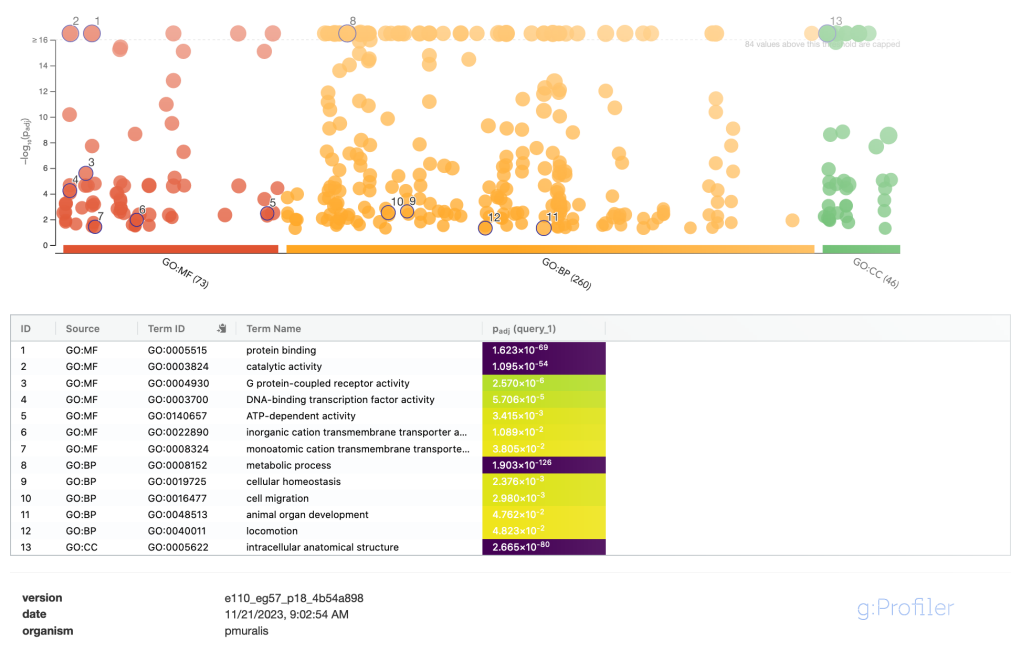

Supplement: Supplementary file 3 — Figure S3. [file ECE3-14-e11407-s005.pdf]

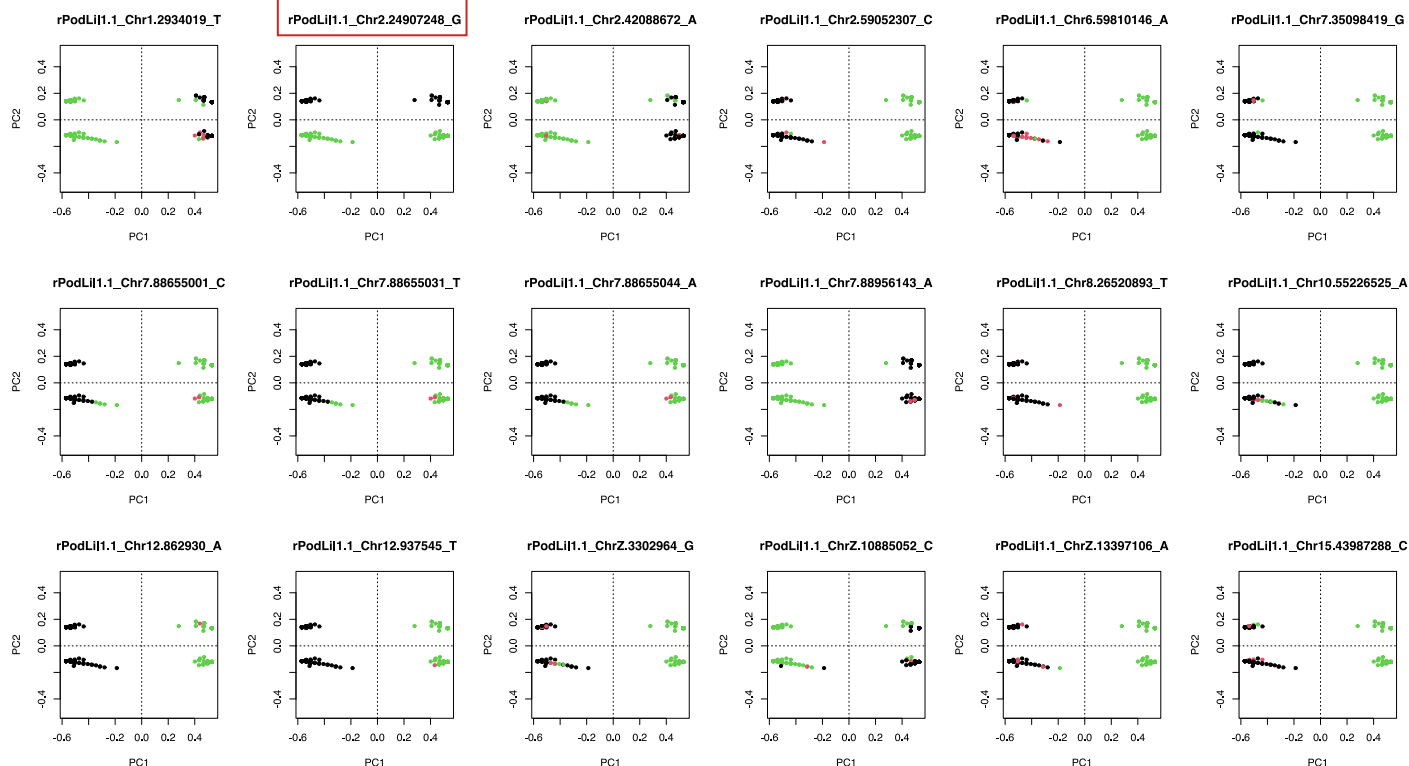

Supplement: Supplementary file 4 — Figure S4. [file ECE3-14-e11407-s001.pdf]
